# Sessile Serrated Lesions: Searching for the True Prevalence and Risk Factors in China

**DOI:** 10.5152/tjg.2024.24188

**Published:** 2024-11-11

**Authors:** Bing-Yue Yao, Li Zhang, Chuan-Xia Wu, Liang Zheng, Ben-Song Duan, Qin-Wei Xu, Jing-Jing Lian, Hai-Bin Zhang, Yu Wang, Jia Cao

**Affiliations:** 1 Department of Gastroenterology, Endoscopy Center, Shanghai East Hospital, School of Medicine, Tongji University, Shanghai, China; 2 Department of Pathology, Shanghai East Hospital, School of Medicine, Tongji University, Shanghai, China; 3 Department of Gastroenterology, Linyi Central Hospital, Linyi, Shandong, China; 4 Heart Failure Research Center, Shanghai East Hospital, School of Medicine, Tongji University, Shanghai, China

**Keywords:** Colonoscopy,, sessile serrated lesions,, detection rate,, risk factors,, colorectal cancer,, retrospective study

## Abstract

**Background/Aims:**

Growing recognition identifies sessile serrated lesions (SSL) as colorectal cancer (CRC) precursors. However, the SSL detection rate remains debatable and lacks a definitive consensus. Additionally, understanding the influencing factors in SSL development is limited. We aim to retrospectively analyze the true prevalence and risk factors of SSL in China.

**Materials and Methods:**

This retrospective study collected medical data from patients who underwent colonoscopy at the Endoscopy Center of Shanghai East Hospital affiliated with Tongji University between March 1, 2019 and February 28, 2022. Data were sourced through the electronic medical record system and included information such as age, sex, lesion location, number, and pathology. This study predominantly focused on the detection rate and the clinical and endoscopic features of SSL.

**Results:**

Of 72 287 colonoscopies in 3 years, 3905 cases were histologically confirmed as SSL. Among them, 2290 (58.6%) were male, and 1615 (41.4%) were female. The overall SSL detection rate was 5.40%, slightly surpassing Asian/Chinese averages but lower than Western rates. Males had a higher SSL detection rate (6.1%) than females (4.6%). Univariate analysis revealed a significant association between SSL with dysplasia/adenocarcinoma (SSL-D/AD) and obesity (Body Mass Index, BMI ≥ 24), CRC family history, and hypertension. After multivariable logistic regression, only obesity (BMI ≥ 24) remained a statistically significant independent risk factor for SSL-D/AD.

**Conclusions:**

The SSL detection rate at our center is 5.4% and increases with age. Males have a significantly higher detection rate than females. Our findings suggest that endoscopists should consider risk factors for SSL-D/AD, such as obesity, CRC family history, and hypertension.

**Main Points**

- This retrospective study offers novel insights into the diagnosis and management of colorectal cancer by analyzing the prevalence and associated risk factors of sessile serrated lesions (SSLs) in a large Chinese population.
- Our findings indicate a slightly higher detection rate of SSLs at an overall 5.40%, compared with China and other Asian countries. Importantly, our comprehensive study is the first to analyze multiple SSLs and examine the risk factors for SSL-D/AD, expanding the current knowledge in this field.
- By providing valuable insights into the detection and management of colorectal cancer, our study highlights the potential significance of optimizing screening strategies for the early detection and treatment of SSLs.

## Introduction

Colorectal cancer (CRC) ranks as the third most prevalent global cancer with high mortality rates.^1-3^ Serrated lesions have been recently recognized as important precancerous lesions, accounting for approximately 30% of CRCs.^4^ In 2019, the world health organization (WHO) reclassified serrated lesions into hyperplastic polyps (HP), sessile serrated lesions (SSL), SSL with dysplasia (SSL-D), traditional serrated adenomas, and unclassified serrated adenomas. Despite this revision increasing SSL awareness, detection rates vary widely among providers and studies. This arises from challenges in visualizing or completely resecting SSLs during colonoscopy, compounded by detection variations among pathologists. Mean SSL detection rates span 2%-10%.^5^

The relationship between SSL, age, and sex sparks debate and warrants further investigation. Additionally, the rapid progression of some lesions to dysplastic or invasive carcinomas underscores the need for investigating the risk factors associated with SSL-D and SSL with adenocarcinoma (SSL-AD). Furthermore, lesions at atypical sites, particularly in the appendix orifice, are frequently overlooked in clinical practice.

Therefore, we conducted a single-center retrospective study to establish the colorectal SSL detection rate and investigate the relationship between age, sex, and SSL. Additionally, this study aimed to identify the risk factors associated with SSL-D and SSL-AD. This study is essential for early cancer prevention through high-risk patient identification.

## Materials and Methods

### Study Protocol and Patients

The clinical data of patients who underwent colonoscopy at the Endoscopy Center of Shanghai East Hospital affiliated with Tongji University between March 2019 and February 2022 were retrospectively collected. The study protocol was approved by the Human Ethics Committee of Shanghai East Hospital affiliated with Tongji University (approval number: 2023049; date: 2023-5-22), and it was conducted in accordance with the Declaration of Helsinki. Written consent was obtained from the legal guardians of the patients to store their information in the hospital database for research purposes. Detailed data were stored in the Endoscopy Center of our hospital and were only accessible with the approval of the patients and the Human Ethics Committee of our hospital.

### Data Extraction

The inclusion criteria were (1) patients who underwent a complete colonoscopy and (2) those with clear pathological and histological diagnoses.

The exclusion criteria were as follows: (1) Patients in whom the colonoscope could not reach the ileocecal portion; (2) Patients who failed to obtain a histopathological diagnosis after polyp removal; (3) Aspirin, clopidogrel, or other anticoagulant/antiplatelet drug intake within 7 days; (4) Inadequate bowel preparation quality (BPQ) (Aronchick score >3); (5) Patients with sessile serrated polyposis, other polyposis, inflammatory bowel disease, etc.; and (6) patients whose pathological type could not be clearly identified or classified.

In this study, we classified individuals aged 18-44 years as “young,” those aged 45-59 years as “middle-aged,” and those aged ≥60 years as “old.” Multiple sessile serrated lesions (SSLs) refer to the presence of more than one SSL in the colorectal region. We defined a smoker as someone who smoked at least 20 pack-years or more regardless of whether he/she quit smoking. Colorectal cancer family history was defined as having at least 1 first-degree relative or 2 second-degree relatives with the disease. Lesion sites were defined as follows: the right colon, including the transverse colon, hepatic area, ascending colon, and cecum; the left colorectum, including the splenic area, descending colon, sigmoid colon, and rectum; and the entire colorectum, including both the right and left colons.

### Colonoscopy

During the colonoscopy, the Japan Olympus 260/290 series and Fujifilm series mirrors were used. The case materials were standardized according to established protocols, and submucosal injection was performed using a mixed solution of normal saline containing 0.4% indigo carmine and 0.025 mg/mL epinephrine. The specimens were fixed using formaldehyde, paraffin embedding, and subjected to section staining for pathological histological examination.

### Diagnosis of SSL (Figure 1)

We had 34 endoscopists perform the colonoscopies, each with more than two years of experience in endoscopic procedures. Following the revised WHO guidelines in 2019, our department has conducted specialized training to enhance the recognition and detection of serrated lesions. A team of 10 pathologists, including specialists in gastrointestinal early cancer pathology, examined the histologic specimens. They received regular professional training on the pathological diagnosis of serrated lesions.

Endoscopic diagnosis: SSLs exhibit diverse endoscopic features, including a mucus cap on white-light endoscopy,^6^ a Red Hat sign observed via narrow band imaging (NBI) endoscopy,^7^ blurred borders and cloudy surfaces on white-light endoscopy,^8^ dilated microvessels,^9^ enlarged crypt openings on NBI,^10^ and type II crypt (type II-O) openings on pigment endoscopy.^11^

Pathological diagnosis: Increased gland diameter and enlarged opening; microbubble-like mucous cells; jagged crypts, widened and inverted crypt base.^12^

### Diagnosis of SSL-D (Figure 2)

Endoscopic morphological findings, such as large or small nodules on the surface and partial protrusion of SSLs, serve as valuable indicators of dysplasia within SSLs.

### Statistical Analysis

Patients diagnosed with SSL were screened, and their clinical characteristics were analyzed and summarized. Univariate analysis was performed using *χ* ^2^ for dichotomous variables and *t-*test for parametric variables. Analyses were performed comparing patients with SSLs to those with SSL-D/AD. Multivariate logistic regression models with age, sex, BMI, and CRC family history as covariates were used to estimate the association between these risk factors and SSL-D/AD. Variables with an initial *P-*value ≤ .05 were entered into a multivariable logistic regression to estimate odds ratios (ORs) and 95% confidence intervals (CIs). Two-sided *P*-values < .05 were considered statistically significant. All statistical analyses were performed using SPSS 26.0 (IBM SPSS Corp.; Armonk, NY, USA).

## Results

### Clinical Characteristics and Detection Rates of SSL

Our study analyzed 3905 cases, with men accounting for approximately 58.6% of the sample. The median patient age was 59 years, with 721 (18.5%) patients aged 18-44 years, 1282 (32.8%) patients aged 45-59 years, and 1902 (48.7%) patients aged ≥60 years. Among the SSLs observed, approximately 88.6% were located in the right colon, 9.7% in the left colon, and 1.7% in the entire colon. Moreover, we found that 1972 (50.5%) polyps were <0.5 cm, 1134 (29.0%) were 0.5-1 cm, and 799 (20.5%) were ≥1 cm. With respect to morphology, we observed 1418 cases of flat polyps, 2170 cases of sessile polyps, and 317 cases of unspecified polyps. These observations align with existing literature reports. Furthermore, our analysis revealed 2970 cases of single SSL and 935 cases of multiple SSLs. Although progression to SSL-D is relatively rare, we observed an incidence of 0.5% for SSL-D, 0.8% for SSL-AD, and 32.3% for SSL with adenoma in our study, as detailed in Table 1.

As presented in Table 2, the study established that the overall detection rate of SSLs was 5.40%. Among the different age groups, individuals aged ≥60 years old had the highest SSL detection rate, reaching 6.7%. The detection rates were 3.4% in the 18-44 year-old age group and 5.6% in the 45-59 year-old age group.

**Figure 3.**
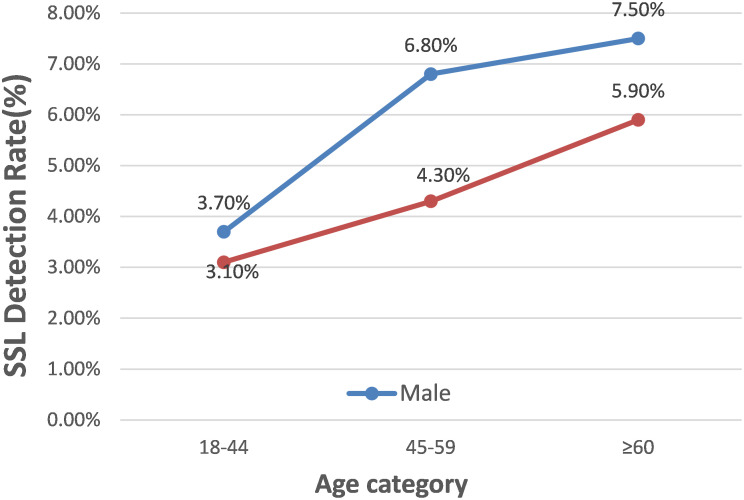
Detection rates of SSLs by various age categories and gender. SSLs: sessile serrated lesions.

Figure 3 illustrated that SSL prevalence increased with age, and males displayed a notably higher SSL detection rate than females (6.1% vs. 4.6%, *P* < .001). This sex difference was consistently observed across different age groups: 3.7% versus 3.1%, 6.8% versus 4.3%, 7.5% versus 5.9% (*P* < .001). Notably, the contrast was most pronounced in patients aged 45-59 years, with an incidence rate of 6.8% in males versus 4.3% in females (*P* < .001).

### Clinical Characteristics and Detection Rates of Multiple SSLs

Table 3 summarizes the correlation between patient age, gender, and the detection rate of multiple SSLs. Overall, the detection rate of multiple SSLs in males was significantly higher than in females (25.2% vs. 22.1%, *P* = .024). In patients aged 18-44 years, the detection rate of multiple SSLs was marginally lower in men than in women, although it did not achieve statistical significance after adjusting for multiple tests (15.5% vs. 16.9%, *P* = .597).

### Risk Factors of SSL-D or AD

Sessile serrated lesions are generally accompanied by dysplasia during carcinogenesis; however, the risk factors for SSL-D/AD remain unknown. As shown in Table 4, in univariate analysis, SSL-D/AD exhibited significant associations with obesity (BMI ≥ 24) (OR: 1.37, 95% CI: 1.12-1.69, *P* = .01), CRC family history (OR: 1.37, 95% CI: 1.12-1.69, *P* = .03), and hypertension (OR: 1.92, 95% CI: 0.98-1.00, *P* = .02). Other factors, including age and smoking status, did not show significant associations with SSLs. Sex (male) (OR: 0.83, 95% CI: 0.63-1.11, *P* = .16), diabetes (OR: 0.85, 95% CI: 0.40-1.81, *P* = .68), and alcohol use (OR: 0.70, 95% CI: 0.35-1.40, *P* = .3) were not associated with the risk of SSL-D/AD.

After multivariable logistic regression, only obesity (BMI ≥ 24) (adjusted OR: 1.12, 95% CI: 1.01-1.26, *P* = .04) remained a statistically significant independent risk factor for SSL-D/AD.

### Clinical Characteristics and Detection Rates of Appendix SSL (Figure 4)

Although reports of SSLs in the appendiceal orifice are limited, our center identified 20 cases of these lesions in this location, as shown in Table 5, offering a valuable foundation for subsequent investigations. Among these 20 patients, eight were male and 12 were female. Additionally, four cases involved patients aged 18-44 years, six involved those aged 45-59, and ten pertained to patients aged ≥60 years. Within this cohort, only 11 affected exclusively affected the appendix, while nine cases extended to other adjoining regions. Furthermore, 7 cases exhibited lesion sizes <0.5 cm, 4 cases ranged from 0.5-1 cm, and 9 cases had sizes ≥1 cm. There were 9 cases of single SSL and 11 cases of multiple SSLs. Lastly, regarding the morphology of the lesions, there were 13 cases with flat polyp morphology, two cases of lateral spreading tumors, and five cases of unspecified polyps.

## Discussion

In our single-center study of screening colonoscopies, we observed an overall prevalence of 5.40% for SSLs, and this prevalence escalates with age and is more pronounced in males. Also, our findings suggest that several factors, including obesity, CRC family history, and hypertension, are associated with an increased risk of SSL progression to SSL-D/AD. The reported detection rates of SSLs in the literature vary widely due to the performance of endoscopists and the accuracy of pathologists in diagnosing these lesions (Table 6). A meta-analysis conducted by Sz-Iuan Shiu et al concluded that the overall prevalence of SSLs was 0.4% (95% CI: 0.2-0.9%) in Asian countries and 4.3% (95% CI: 3.0-6.1%) in Western countries.^13^ Another meta-analysis found that the average detection rate of SSLs worldwide ranged from 2%-8%.^5^ Notably, in a large retrospective study conducted by Chen et al in southern China, the incidence of serrated polyps/adenomas was 0.2%,^14^ while another domestic single-center large sample regression study found that the detection rate of serrated lesions during colonoscopy was 0.7%.^15^ These studies consistently show that the detection rate of SSLs in China is significantly lower than in Western countries. However, our study’s detection rate of 5.4% is significantly higher than the reported average detection rate in China and other Asian countries, and it is similar to that of Western countries.^13^

It is important to note that there are significant variations in SSL detection rates among endoscopists and examination centers.^5,16,17^ Analyzing our center’s data, we found that 11 endoscopists detected SSLs in 2019, increasing to 14 in 2020, and further rising to 34 in 2021. Our analysis revealed two main reasons for this significant improvement: Firstly, after the WHO revised the fifth edition of the colorectal-serrated lesions definition in 2019, specialized learning and regular training programs were implemented. These initiatives have provided young doctors with a better understanding of serrated lesions, increasing their sensitivity in detecting such lesions. Consequently, the detection rate among young doctors has gradually increased. Secondly, since the arrival of a renowned domestic pathologist specializing in early digestive tract cancer at our hospital in 2020, regular professional training programs for pathologists on the diagnosis of serrated lesions have been conducted. This has greatly contributed to the sharp increase in the detection rate of SSLs at our center.

The relationship between SSL, age, and sex remains a subject of debate. A large retrospective analysis conducted in the United States.^5^revealed that the prevalence of SSLs increased with age. Our results were consistent with this conclusion (Figure 3). However, some studies have reported that age is not a factor affecting the incidence of SSLs.^18^ Studies investigating sex-based differences in SSL detection have yielded conflicting results. Sanaka et al found that the incidence of adenoma was higher in males than females, and there was no significant difference between males and females in SSL incidence.^19^ In contrast, Alvarez et al found that men had a higher incidence of SSLs than women.^20^ Furthermore, Teriaky et al found that the incidence of SSLs was higher in women than in men.^21^ Our study found that the rate of SSL detection was significantly higher in males than females, with this sex difference being most significant in patients aged 45-59 years, with an incidence rate of 6.8% in males and 4.3% in females (*P *< .001). Our clinical experience suggests that young females are more prone to developing multiple SSLs. To investigate this observation, we conducted statistical analyses of the collected data. Among patients aged 18-44 years, the detection rate of multiple SSLs in females was slightly lower than that in males, although this difference was not statistically significant after multiple test corrections (0.6% vs. 0.5%, *P* = .586). However, this sex gap was much smaller than that in other age groups, indicating partial consistency with our clinical experience. These inconclusive results may be attributed to the limited number of eligible patients. Therefore, more multicenter or large-scale studies are required to further investigate this issue and validate our findings.

Serrated polyps have been considered benign lesions in the past and do not have malignant tendencies; however, in recent years, the serrated carcinogenesis pathway has attracted widespread attention as a new colon carcinogenesis pathway. SSLs are important precancerous lesions for CRC. Studies have shown that the average progression time from SSL to SSL-D is approximately 17 years.^22^ However, once SSL-D develops, it rapidly progresses to invasive carcinoma. Compared to traditional tubular adenomas, SSLs are associated with a higher likelihood of lymphatic invasion and lymph node metastasis in submucosal infiltrating cancers.^23^ Therefore, early identification of SSLs plays a crucial role in preventing CRC and especially “interval cancer.” Many studies have investigated the risk factors associated with SSL. The available literature suggests that white ethnicity, CRC family history, and personal history of precancerous serrated polyps are consistent risk factors for SSL in studies conducted in Western countries.^21^ Nevertheless, few studies have investigated risk factors associated with SSL-D or SSL-AD. In our study, on univariable analysis, SSL-D/AD was significantly associated with obesity (BMI ≥ 24) (OR: 1.37, 95% CI: 1.12-1.69, *P* = .01), CRC family history (OR: 1.37, 95% CI: 1.12-1.69, *P *= .03), and hypertension (OR: 1.92, 95% CI: 0.98-1.00, *P* = .02). After multivariable logistic regression, only obesity (BMI ≥ 24) (adjusted OR: 1.12, 95% CI: 1.01-1.26, *P* = .04) remained a statistically significant independent risk factor for SSL-D/AD. The results showed that SSL-D/AD detection was significantly more likely in patients with obesity. Our findings support previous studies that have demonstrated that older age, male sex, and obesity are associated with increased SSL^23-25^ and that SSL can rapidly progress to SSL-D/AD.

Sessile serrated lesions located in the appendix orifice possess a certain incidence rate; however, they are often overlooked. To address this issue, our center mandates that every endoscopist comprehensively explore this area when performing endoscopies on patients. Our center identified 20 cases of SSLs in the appendix orifice, a relatively uncommon location for this type of lesion, which serves as a valuable reference for future studies.

Our study had several strengths that enhanced the validity and relevance of the findings. First, we used a large and well-characterized population, which enabled us to comprehensively investigate the detection rates and risk factors associated with SSLs. Second, we relied on the histopathological descriptions of the polyps, which improved the accuracy of our results. Third, the high photo-documented cecal intubation rate and reasonably high-quality bowel preparations enhanced the reliability of our data. Additionally, this comprehensive study, to the best of our knowledge, is the first to analyze multiple SSLs and examine the associated risk factors for SSL-D/AD, thus providing novel insights into the diagnosis and management of CRC.

The retrospective design of our study is limited by missing or inconsistently reported data (such as smoking and alcohol use) and unknown biases, and our data collection was not based on random sampling, which could introduce potential biases. Further high-quality, large prospective studies are required to address these limitations.

In conclusion, our research provides critical insights into the prevalence of SSL, highlighting a detection rate of 5.4% that escalates with age and is more pronounced in males. Overall, our study contributes significantly to the current understanding of the prevalence and risk factors associated with SSLs, highlighting the need to optimize screening guidelines for the early detection and management of CRC.

## Figures and Tables

**Figure 1.**
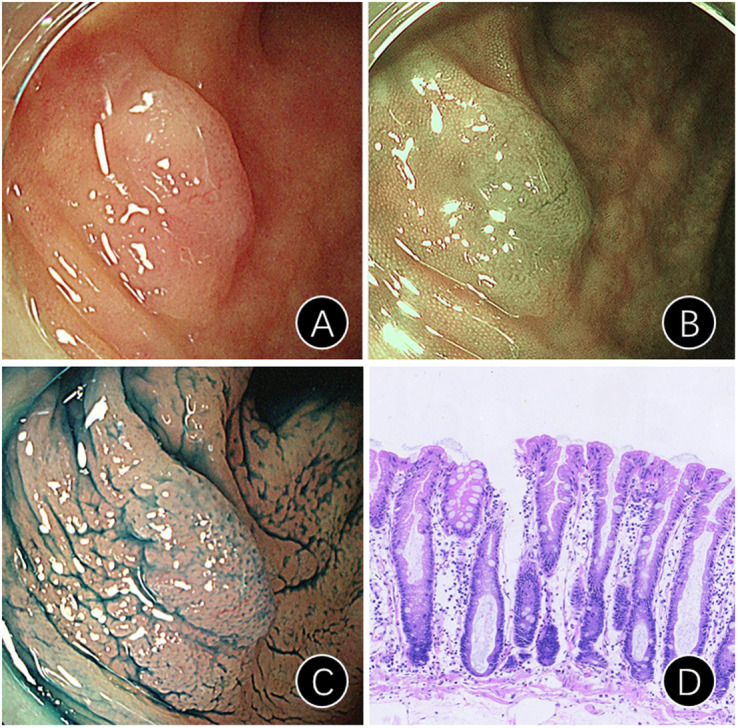
Typical endoscopic and pathological images of SSL patients. Pictures A-D are SSL with white endoscope, NBI, Indigo rouge staining, and pathology (HE staining ×100) images; NBI: Narrow band imaging; SSL: Sessile serrated lesions.

**Figure 2.**
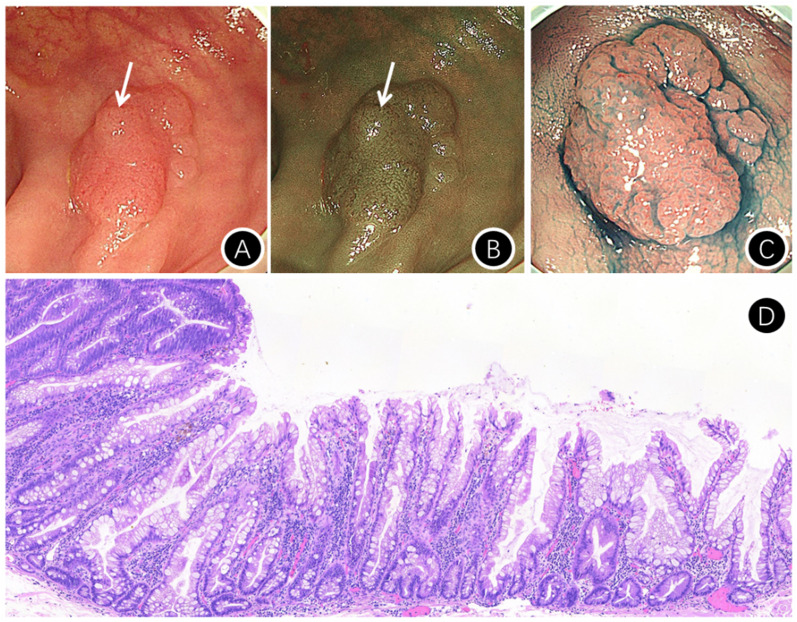
Endoscopic features of sessile serrated lesion with dysplasia. A: Small nodule on the lesion surface (arrow) [white light endoscopy]; B: Small nodule on the lesion surface (arrow) [narrow-band imaging (NBI) endoscopy]; C: [Indigo rouge staining]; D: Pathology (HE staining ×100) images of SSL-D.

**Figure 4.**
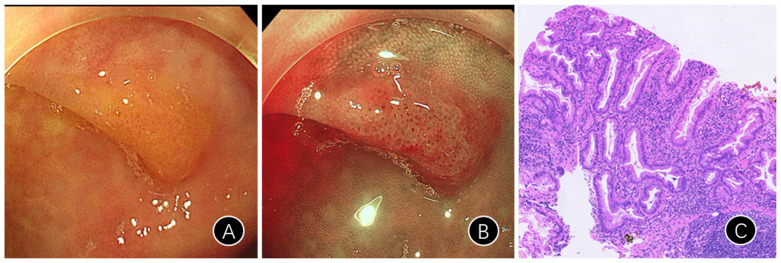
Typical endoscopic and pathological images of patients with SSL in the appendix fossa. A: Mucous cap (white light endoscopy); B: Red cap sign (narrow-band imaging [NBI] endoscopy); C: Pathology (HE staining ×100) images of the SSL in the appendix fossa. SSL: sessile serrated lesions.

**Table 1.** Clinical and Pathological Features of SSL in Patients

	SSL
Total	3905
Sex	
Male	2290 (58.6%)
Female	1615 (41.4%)
Age	
18-44	721 (18.5%)
45-59	1282 (32.8%)
≥60	1902 (48.7%)
Location	
Right	3461 (88.6%)
Left	380 (9.7%)
Whole	64 (1.7%)
Size	
<0.5 cm	1972 (50.5%)
0.5-1 cm	1134 (29.0%)
≥1 cm	799 (20.5%)
Morphology	
Flat	1410 (36.1%)
Sessile	2216 (56.7%)
Missing	279 (7.2%)
Total number of SSL	
1	2970 (76.1%)
>1	935 (23.9%)
Other pathological types	
SSL with dysplasia	21 (0.5%)
SSL with adenocarcinoma	30 (0.8%)
SSL with adenoma	1261 (32.3%)

**Table 2.** Associations Between Individual Patient Cohort Factors and SSL Detection

Patientcohort Factor	Total	SSLs		*P-Value*
		*Yes *(*n = 3905*)	*No *(*n = 68 382*)	
Sex		**3905** (**5.4%)**		<.001
* Male*	37 318 (51.6%)	2290 (6.1%)	35 028 (93.9%)	
* Female*	34 969 (48.4%)	1615 (4.6%)	33 354 (95.4%)	
Age categories				
18-44		**721 **(**3.4%)**		.01
* Male*	11 303 (53.5%)	420 (3.7%)	10 883 (96.3%)	
* Female*	9816 (46.5%)	301 (3.1%)	9515 (96.9%)	
45-59		**1282 **(**5.6%)**		<.001
* Male*	11 772 (51.8%)	806 (6.8%)	10 966 (93.2%)	
* Female*	10 944 (48.2%)	476 (4.3%)	10 468 (95.7%)	
≥60		**1902 **(**6.7%)**		<.001
* Male*	14 243 (50.1%)	1064 (7.5%)	13 179 (92.5%)	
* Female*	14 209 (49.9%)	838 (5.9%)	13 371 (94.1%)	

**Table 3.** Associations Between Individual Patient Cohort Factors and Multiple SSL Det ection

Patient Cohort Factor	Total SSLs	Multiple SSLs	Single SSLs	*P*-Value
	(n = 3905)	(n = 935)	(n = 2970)	
Sex				**.024**
Male	2290 (58.6%)	578 (25.2%)	1712 (74.8%)	
Female	1615 (41.4%)	357 (22.1%)	1258 (77.9%)	
Age categories (years)				
18-44				.597
Male	420 (3.7%)	**65 **(**15.5%)**	355 (84.5%)	
Female	301 (3.1%)	**51 **(**16.9%)**	250 (83.1%)	
45-59				**.012**
Male	806 (6.8%)	216 (26.8%)	590 (73.2%)	
Female	476 (4.3%)	98 (20.6%)	378 (79.4%)	
≥60				.129
Male	1064 (7.5%)	297 (27.9%)	767 (72.1%)	
Female	838 (5.9%)	208 (24.8%)	630 (75.2%)	

**Table 4.** Comparison of Patient Characteristics Among Sessile Serrated Lesion (SSL) and SSL with Dysplasia or Adenocarcinoma (SSL-D/AD) Group n (%)

	SSL-D/AD (n = 51)	SSL (n = 3854)	Univariate OR (95% CI)	*P*-Value	Multivariate OR (95% CI)
Sex (male)	25 (49.0%)	2265 (58.8%)	0.83 (0.63-1.11)	.16	
Age (year) (≥median)	29 (56.9%)	1873 (48.6%)	1.17 (0.92-1.49)	.24	
Obesity (BMI) (≥24)	33 (64.7%)	1816 (47.1%)	1.37 (1.12-1.69)	**.01**	1.12 (1.01-1.26) **(*P*** = **.04)**
CRC family history	6 (11.8%)	177 (4.6%)	2.57 (1.19-5.50)	**.03**	0.35 (0.09-1.33) (*P* = .13)
Diabetes	6 (11.8%)	532 (13.8%)	0.85 (0.40-1.81)	.68	
Hypertension	21 (41.2%)	1021 (26.5%)	1.92 (0.98-1.00)	**.02**	0.74 (0.35-1.56) (*P* = .43)
Smoking	8 (15.7%)	527 (13.7%)	1.15 (0.60-2.18)	.68	
Alcohol use	7 (13.7)	753 (19.5)	0.70 (0.35-1.40)	.3	

**Table 5.** Clinical and Endoscopic Features of 20 Patients with Appendix SSL

	SSL
Sex	
Male	8
Female	12
Age (years)	
18-44	4
45-59	6
≥60	10
Location	
Appendix	11
Appendix with other part	9
Size	
<0.5 cm	7
0.5-1 cm	4
≥1 cm	9
Total number of SSL	
1	9
>1	11
Morphology	
Flat	13
LST	2
Null	5

**Table 6.** A Summary of Studies Concerning SSL Detection Rate

Study	Year	Region	Sample Size	Mean Age (Years)	Male (%)	SSL Detection Rate (%)
Vidit et al^26^	2022	Australia	2091	54	53.4	13.8
Zhou et al^27^	2020	USA	10 538	60	42.8	2.2
Rashid et al^28^	2021	China	6011	60	53.7	1.4
Liu et al^15^	2018	China	38 981	57.8	57	0.18
Davenport et al^29^	2018	USA	6404	57.8	64	3.34
Maratt et al^30^	2017	USA	2416	61.4	61	7.86
Cao et al^31^	2016	China	28 981	60.3	63.6	0.038
Saiki et al^32^	2016	Japan	15 326	65.7	68.6	4
Yang et al^33^	2015	USA	11 201	61	46.3	5.02

## Data Availability

The data that support the findings of this study are available on request from the corresponding author.
