# Erratum

**DOI:** 10.5152/tjg.2025.030625

**Published:** 2025-01-31

In the original article authored by Yao et al, titled “Sessile Serrated Lesions: Searching for the True Prevalence and Risk Factors in China” (Turk J Gastroenterol. 2025; 36(1): 15-23. DOI: 10.5152/tjg.2024.24188) published in the January 2025 issue of Turkish Journal of Gastroenterology, the declaration regarding the funding was written incorrectly and the statement that the first three authors contributed equally to the article has not been included by an oversight. Subsequently, the correct funding statement and author contributions added, and the online version of the original article has been updated accordingly. You can access the revised version of this original article via the following link:

https://www.turkjgastroenterol.org/en/sessile-serrated-lesions-searching-for-the-true-prevalence-and-risk-factors-in-china-137294

